# Controlling grain structure in metallic additive manufacturing using a versatile, inexpensive process control system

**DOI:** 10.1038/s41598-023-37089-x

**Published:** 2023-06-20

**Authors:** Lova Chechik, Alexander D. Goodall, Katerina A. Christofidou, Iain Todd

**Affiliations:** Department of Materials Science and Engineering, The University of Sheffield, Mappin Street, Sheffield, S1 3JD UK

**Keywords:** Metals and alloys, Laser material processing

## Abstract

Additive manufacturing (AM), commonly termed 3D printing, is a revolutionary manufacturing technology with great industrial relevance in the aerospace, medical and automotive sectors. Metallic AM allows creation of complex intricate parts and repair of large components; however, certification is currently a concern due to lack of process consistency. A versatile, inexpensive process control system was developed and integrated, reducing variability in melt pool fluctuation and improving microstructural homogeneity of components. Remnant microstructural variation can be explained by the change in heat flow mechanism with geometry. The grain area variability was reduced by up to 94% at a fraction of the cost of a typical thermal camera, with control software written in-house and made publically available. This decreases the barrier to implementation for process feedback control, which can be implemented in many manufacturing processes, from polymer AM to injection moulding to inert-gas heat treatment.

## Introduction

Laser directed energy deposition (L-DED) is a type of metallic additive manufacturing, which enables the deposition of larger components and may also be used for repair of components because of its ability to deposit on a previously existing part e.g. repairing turbine blisks^1,2^. The applicability of L-DED to component repair has been widely reported^3^, however process consistency is still lacking^1^, limiting the uptake of the technology within industries necessitating high performance components. Consequently, there is a need for adaptive process control in L-DED to achieve accurate component geometries and eliminate defects such as porosity^4^. Certification issues are reported as being the main barrier to L-DED being more widely integrated in the aerospace industry^2,5^. If the process were better controlled, this could improve component reliability and process control could become a part of the certified manufacturing method^2^.

Process control has been widely used in industry to directly control values such as temperature, pressure and fluid flow rate within manufacturing and post-manufacturing operations^6,7^. For example, proportional-integral-derivative (PID) controllers are widely used to maintain quality and consistency in production lines^6^. Process control is difficult in L-DED as there is no obvious value that must be kept constant to produce a homogenous component. The mechanical properties of a metal are dependent on the scale of the microstructure (including grain size, dendrite arm spacing and precipitate dispersion) and the heat treatments experienced^8^. The scale of the microstructure is dependent on the cooling rate during solidification, which can be correlated with the melt pool size^9,10^. This leaves many values which could be controlled to improve component homogeneity.

Process control using temperature as a target is most common in L-DED, using either photodiodes or pyrometers to measure a temperature and adjusting towards a pre-set target temperature^11–14^. Similarly, cameras can be used to measure the melt pool directly, typically coaxially. These measure the melt pool dimensions e.g. area or width, and control the process using this value^15,16^. The metallic microstructure is closely linked to the cooling rate, which has recently been used as the control variable by Farshidianfar et al.^17^. Monitoring frequently uses expensive hardware e.g. thermal cameras made by FLIR, Optris and Jenoptik, that range in the price from $4000 to $30,000.

In L-DED, two variables are commonly adjusted to control the process, these are the laser power^13,15,18^ and velocity^17,19,20^, commercial solutions are available, but are often provided as a black-box from the manufacturer, with limited versatility. Proportional integral derivative (PID) controllers are often used, but these require numerous runs to optimise gain constants^15,19^. The nature of the controller determines how quickly the target is achieved and the magnitude of overshooting.

The vast majority of work in the field of process control in L-DED focusses on analysing the controller performance and the resultant thermal trends^11,15,21–25^. The analysis is often in the form of qualitative results^15,17,19,21,26^, where further benefit could be gained by quantifying data. Most studies omit analysis of components built without control (with constant processing parameters)^13,19,21,26^, thus conclusions on the effectiveness of proposed control systems cannot be easily derived. Hofman et al.^15^ demonstrate that by controlling laser power to keep a constant melt pool width, the hardness variation along the component length can be reduced. Farshidianfar et al.^17^ show micrographs for both open loop (no control) and closed loop (velocity control) but with no clear trends visible or quantified.

The work presented herein investigates whether the pixel-wise sum of a coaxial image, captured by a low/cost camera, can be effectively used for process control. The influence and limitations of process control on microstructural homogeneity is explored, with 2 microstructural measures quantified using EBSD analysis. Further the effect on hardness is measured as a simple proxy for mechanical properties.

## Results and discussion

### Processing parameters and thermal monitoring

Process control is widely researched in the field of AM due to the variability in the process currently experienced. For a variety of reasons such as component geometry, powder size distribution and heat accumulation, even if a component is printed many times with identical processing parameters (e.g. power, velocity), there will be significant scatter in the mechanical properties of the final component. The idea behind process control is as follows:

- consistent and predictable mechanical properties are desired
- control of microstructure can yield the desired consistency in achieved mechanical performance
- component microstructure is dependent on the thermal conditions in the process

It follows that if the thermal conditions are kept constant, then one would expect the microstructure to be homogenous. This would in turn reduce the variability in the mechanical properties of the component.

The difficulty is defining “thermal conditions”; provisional results show thermal intensity (sum of pixels in the image) to be well correlated with both maximum intensity and melt pool area. As such, the thermal intensity was used as the measure of “thermal conditions” to be controlled, similarly to Baraldo et al.^23^.

**Figure 1:**
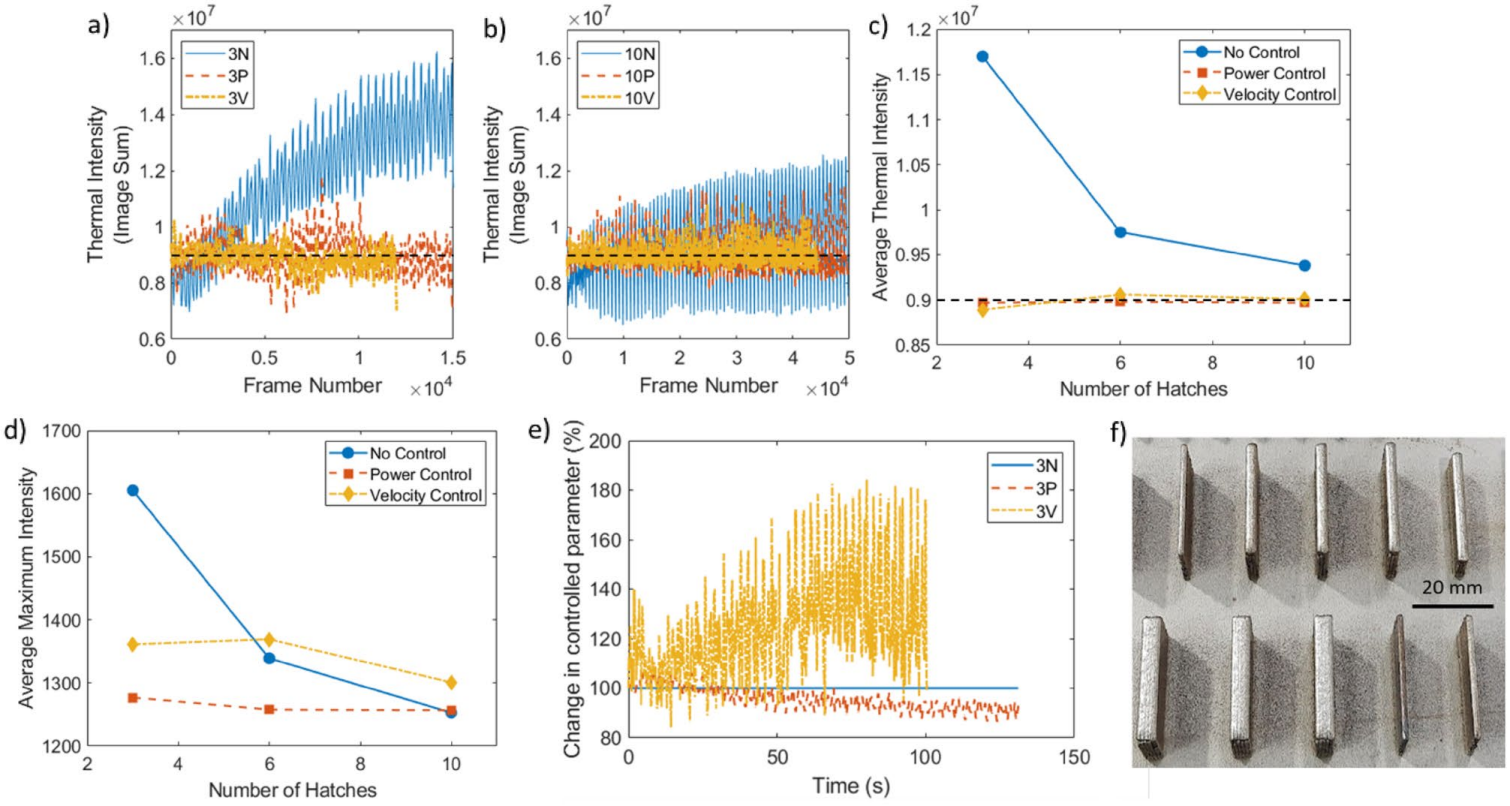
Influence of feedback control on processing parameters and the resulting thermal properties. (**a**, **b**) Thermal intensity of during builds of 3 and 10 hatch walls, respectively (100 point moving mean applied), target value shown in black; (**c**, **d**) effect of process control on thermal intensity and maximum intensity, respectively. Due to the number of data points, the largest standard error for thermal intensity was 2.5 × 10^4^ and for maximum intensity, this was 3, neither of which would be visible on the plot; (**e**) required change in processing parameter by control algorithm for 3 hatch wall; (**f**) representative image showing a range of samples, with different widths and types of process control. N, no control; P, power control; V, velocity control.

To investigate process control, a build with no control (i.e. constant processing parameters, labelled N) was compared directly with a controlled build (power or velocity control, labelled P and V respectively). Once power or velocity control were introduced (corrections performed at an interval of 0.15 s), the power/velocity values were adjusted to target a thermal intensity of 0.9 × 10^7^. Initial processing parameters and the thermal intensity target were both selected to produce components which appeared satisfactory, as exemplified in Fig. 1f.

Figure 1a shows the variation for 3 hatch walls; with no control, the thermal intensity increases with time, appearing to plateau near the end of the build, this has been reported in literature^15,21,27^. For both power and velocity control, the thermal intensity is maintained near the target of 0.9 × 10^7^ (shown in black). Figure 1b shows a similar trend for the 10 hatch wall, however, the wider walls show a smaller increase in thermal intensity through the build (with no control). Process control reduced the standard error of thermal intensity by 45% in the 3 hatch walls and by 25% in the 10 hatch walls. This is definitive evidence that the process control achieved its desired function—the thermal intensity was brought to the target value and the variation of thermal intensity was greatly reduced.

Figure 1c shows how well the controlled walls hit the target, irrespective of wall thickness; it is interesting to note that the natural variability in the 3 hatch wall is much larger than that of the 10 hatch wall. Particularly, the thermal intensity changes more with height in these narrower walls and the thermal intensity reaches greater values, showing that the narrower walls are further from thermal equilibrium at the start of the process. Overall, the power control samples showed average thermal intensity values closest to the target value. Figure 1d shows that the maximum intensity follows the same trend as the thermal intensity (Fig. 1c); the control function was not controlling maximum intensity directly, indicating that the overall morphology of the melt pool was controlled.

Figure 1e shows the processing parameters used for the 3 hatch walls; for walls without control (blue), the parameters were constant, staying at 100% for the duration of the build. The power control walls (orange) show that the power required to hit the thermal intensity target was lower than the pre-set value, reducing to 91% for the 3P wall. The velocity control walls (yellow) show that the control loop increased velocity to ~ 129% to keep thermal intensity constant. Since the velocity of the laser was increased, the processing time was decreased; this could be useful in industrial applications, where a reduced manufacturing time would lead to a reduction in cost. Similar processing parameter trends are seen for wider walls, but with smaller parameter changes required (3% power decrease and 20% velocity increase respectively). A greater percentage change in velocity is needed to control the thermal intensity (compared to power change required), demonstrating that the L-DED process is more sensitive to laser power than laser velocity.

By implementing process control on a complex shape build, the required change in parameters (for a constant thermal signature) could be experimentally determined. This could then be used as a feed-forward control system, using these newly calculate processing parameters to build this specific geometry on different machines. This would result in a more homogenous temperature distribution in the components without requiring every machine to have thermal monitoring capabilities. The reactive aspect of process control would be lacking, but this would be a quantitative method of determining variable processing parameters for new geometries.

### Microstructural analysis

**Figure 2:**
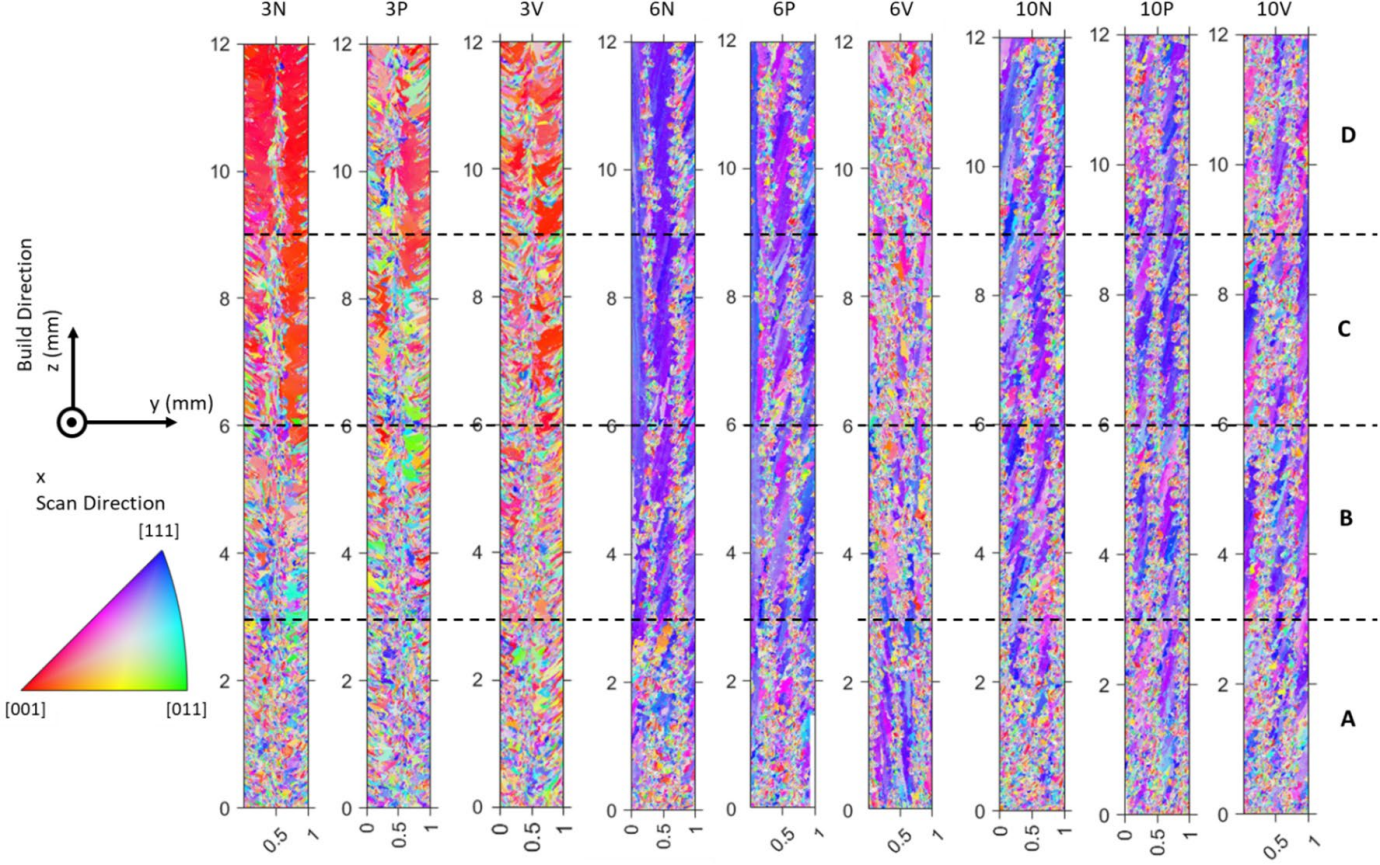
Inverse pole figure maps (IPF-X) of control walls. Four quadrants (**A**–**D**), labelled with height; axis directions shown.

Grain orientation maps for the control walls are shown in Fig. 2, with the full 12 × 1 mm region shown. The 3 hatch walls all have small, randomly oriented grains near the baseplate, but with height, the grain size increases. The 3N wall has some very large, red grains (<100> orientation) towards the top of the sample; along the centre in the y direction, there are fine grains along the full height of the build—these are at the laser centreline. The 3P and 3V orientation maps show similar trends, but with smaller grains retained towards the top.

**Figure 3:**
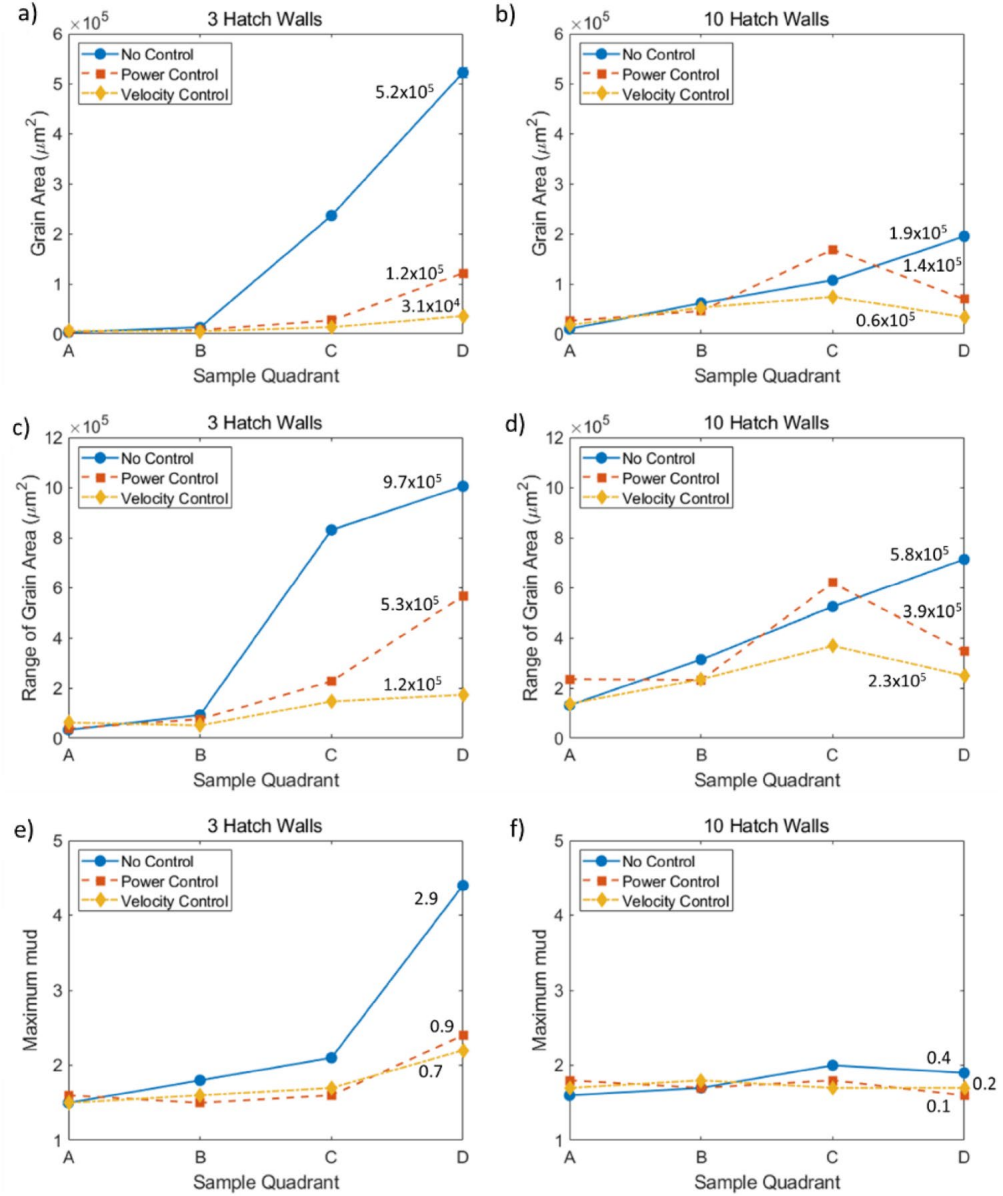
Variation of microstructure with component height, as calculated from EBSD data. (**a**, **b**) area-weighted average grain area; (**c**, **d**) range of grain areas; (**e**, **f**) maximum mud, no errors included as maximum values selected; (**a**, **b**) 3 hatch wall; (**c**, **d**) 10 hatch wall. Lines labelled with the variability for each sample (maximum quadrant value−minimum quadrant value).

For the 6 and 10 hatch walls, a near <112> orientation is dominant, as demonstrated by the primarily blue/purple colour of the IPF-X maps in Fig. 2. For both widths, in both N and P walls, tall, elongated grains occur early on, with small grains along the centrelines. In 6 V and 10 V, these tall, elongated grains are less dominant; instead, a large number of small, randomly oriented grains are retained higher up the wall. To quantify these changes, the walls were split along their height into quadrants, labelled A–D (bottom–top). Figure 3 shows that the narrow wall (3 hatch) experiences the biggest changes with height, both in terms of grain area (Fig. 3a and c) and in terms of maximum mud (Fig. 3e, a greater maximum mud signifies a stronger texture), the combination of these two factors will be used to generally describe the microstructure.

Walls with no control experience the largest range in microstructure (both grain area and maximum mud), with both power and velocity control decreasing this variability in microstructure with height; for each measure, the variability of each line is labelled line and is defined as the minimum quadrant subtracted from the maximum quadrant. Figure 3a and b show that velocity control leads to the greatest reduction in grain area variability with height; with 3 V reducing grain area variability by 94% when compared to 3N. Due to the extent to which the data is skewed (grain area varying by almost 5 orders of magnitude), weighted standard deviation values are nonsensical, being larger than the range; as such, the range of grain areas for each quadrant was also presented (Fig. 3c and d). Identical trends in grain area variability were shown by this measure, with velocity control having the strongest effect on grain area homogenisation—confidence in this result is improved because two different calculations yield the same conclusion.

Process control also results in a decrease in maximum mud variability visible (Fig. 3e and f) and it seems velocity control has a greater effect than power control (up to a 76% reduction in variability). The variability in the wider walls was also reduced, albeit by a smaller margin (70% reduction in grain area variability and 75% reduction in maximum mud variability). In both cases, velocity control resulted in a more significant decrease in microstructural variability.

### Remnant microstructural variability

Despite the increase in microstructural homogeneity (both grain area and maximum mud) observed due to process control, the 3 hatch walls have a predominantly < 100 > grain orientation, whilst wider walls (6 and 10 hatches) predominantly contain grains of < 112 > orientation. As we were controlling the melt pool intensity to the same value for each wall width, we would have expected microstructures to be comparable, but this was not the case, even for walls with no control. In order to gain an understanding of this change in grain orientation, walls of 1–8 hatches were built without process control (Table 1).

**Table 1:** Processing parameters used for walls of varying thickness.

Sample	Sample width (mm)	Power (W)	Velocity (mm/min)	Hatch spacing (µm)	Z Step (µm)	Mass flow (g/min)
1 Hatch wall	1.1	300	2250	400	200	6–6.5
2 Hatch wall	1.3					
3 Hatch wall	1.7					
4 Hatch wall	2.1					
6 Hatch wall	2.8					
8 Hatch wall	3.6					

**Figure 4:**
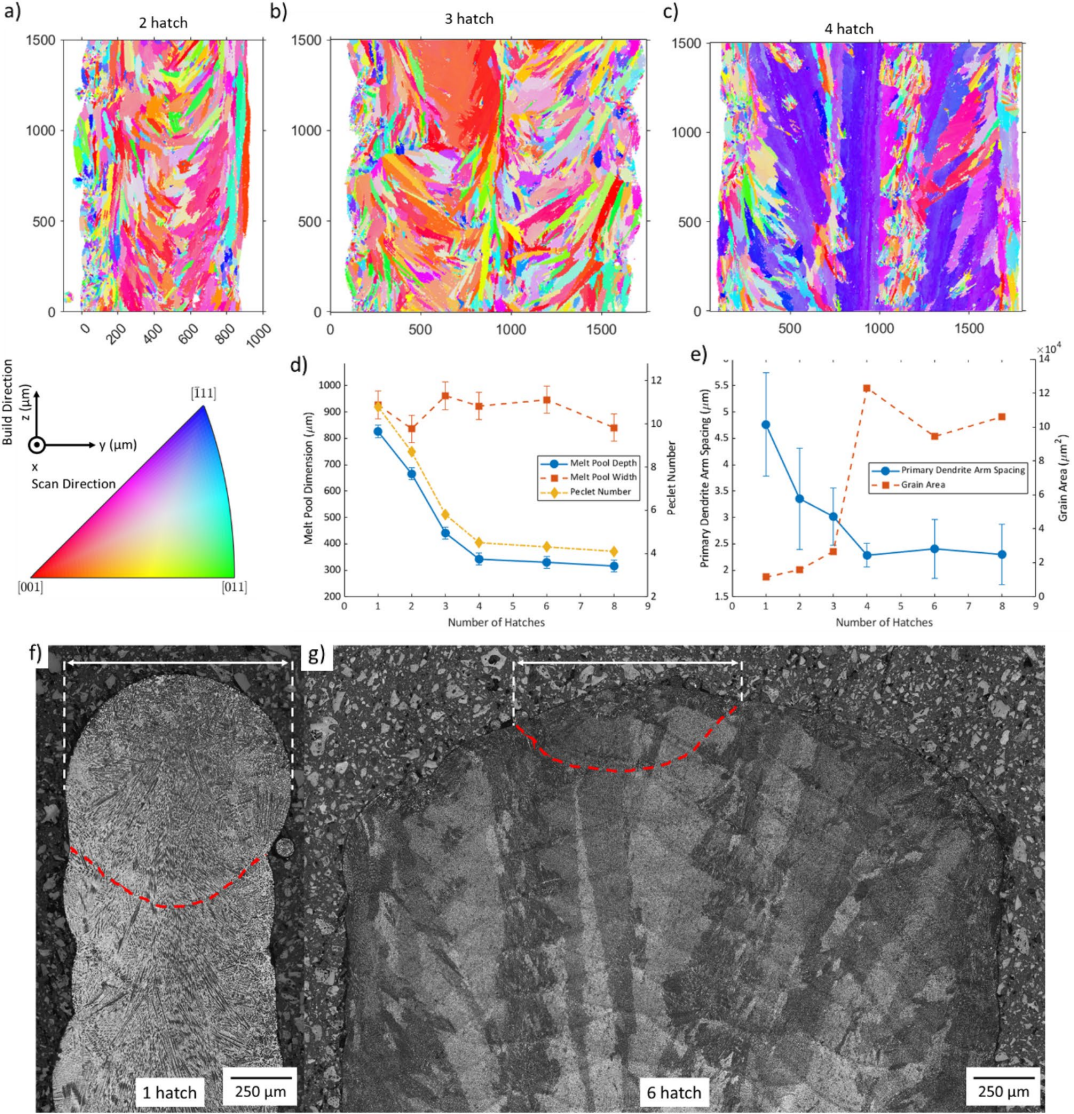
Changes in sample microstructure with varying thickness. (**a**–**c**) inverse pole figure (IPFX) maps of walls from 2 to 4 hatches built with no control; axis definition and legend included; (**d**) melt pool depth, width and Peclet number for samples with increasing number of hatches, error bars are representative standard deviation of melt pool dimensions (as calculated in the 6 hatch sample); (**e**) change in primary dendrite arm spacing (standard deviation shown) and grain area of full mapped region with number of hatches; (**f**, **g**) YZ optical micrographs of 1 and 6 hatch samples, showing the outline and width of a melt pool.

Microstructural analysis of these new walls (Fig. 4a, b and c) shows that this is a sharp transition occurs between 3 and 4 hatches (roughly 2 mm width), explaining why we previously observed a change between 3 and 6 hatches.

To further investigate this phenomenon, the melt pool size and primary dendrite arm spacing of walls of varying thickness were measured optically. The melt pool width and depth for 6 walls of varying numbers of hatches are shown in Fig. 4d, with example micrographs shown in Fig. 4f and g. The melt pool width is relatively consistent (~ 900 µm) for walls of varying thickness, whilst the melt pool depth decreases significantly with increasing sample width until 4 hatches wide (from 830 to 340 µm), at which point the melt pool depth stabilises. Since, narrow walls have much deeper melt pools, but similar XY sections, it can be concluded that the melt pool volume is much larger.

Literature shows that the larger a melt pool, the slower the cooling rate^9,10^; despite using constant processing parameters, it seems that the component geometry is greatly affecting the melt pool depth and so very different thermal conditions are being experienced. These observations are in line with the calculations made by Vasinonta et al.^28^, who observed that for constant processing parameters, the melt pools were much longer in a thin walled component when compared to a bulk component. Assuming that the wide walls (shallow melt pools) have higher cooling rates^9,10^, this would increase the thermal gradient and result in a more columnar microstructure^29^. This aligns with the observations from the EBSD analysis; wider walls show a greater tendency for tall columnar grains, whilst narrow walls mainly experience smaller grains of a more equiaxed nature.

The Peclet number represents the influence of convection in the process (Fig. 4d), using the melt pool depth as the characteristic length; the narrow walls have a larger Peclet number, suggesting that they experience more convection than wider walls. This high Peclet number is indicative of a low Fourier number, confirming that more heat is being stored than is being diffused away, especially in narrow walls^30^; this explains the longer duration taken for a temperature plateau to be reached in narrow walls (Fig. 1a). Once the width of the wall exceeds 2 mm, conduction becomes more significant and a bulk steady-state is reached as sufficient heat can be dissipated.

In literature, Peclet number is shown to scale with energy density^31^ and it is reported that laser velocity is a key factor in determining the convection behaviour^32^. The general approach is that we control how much heat enters the component by setting the processing parameters, this changes the melt pool morphology and the Peclet number, which determines the heat leaving the component. However, it has been shown that the heat flow properties of the component can be varied by changing the geometry. This means that whilst using constant parameters, a 2.5 × change in Peclet number can be observed, simply by altering the component thickness. As well as being able to change the Peclet number by altering the heat input, it may also be altered by changing the geometry, significantly influencing the heat flow mechanisms within the component. Since geometry is normally fixed, the heat flow conditions must be better understood to allow for process control that can overcome this change in heat flow mechanism.

Primary dendrite arm spacings (PDAS) were also measured, and are plotted in Fig. 4e. Narrower walls were found to have a greater PDAS than wide walls, however, from 4 hatches upwards, there was little change in the scale of the microstructure. The grain area, Fig. 4e, shows an inverse relationship when compared to PDAS, with wider walls experiencing larger grain areas, this can be explained by the large columnar nature of the grain structure in the wider walls, once again, there seems to be little change in grain area in samples greater than 4 hatches wide. Both of these results suggest that in wider walls, the cooling rate no longer changes significantly, it can be concluded that at this width, the sample structure is no longer dependent on the width, so the sample can be considered bulk, as demonstrated by the stabilisation of Peclet number.

**Figure 5:**
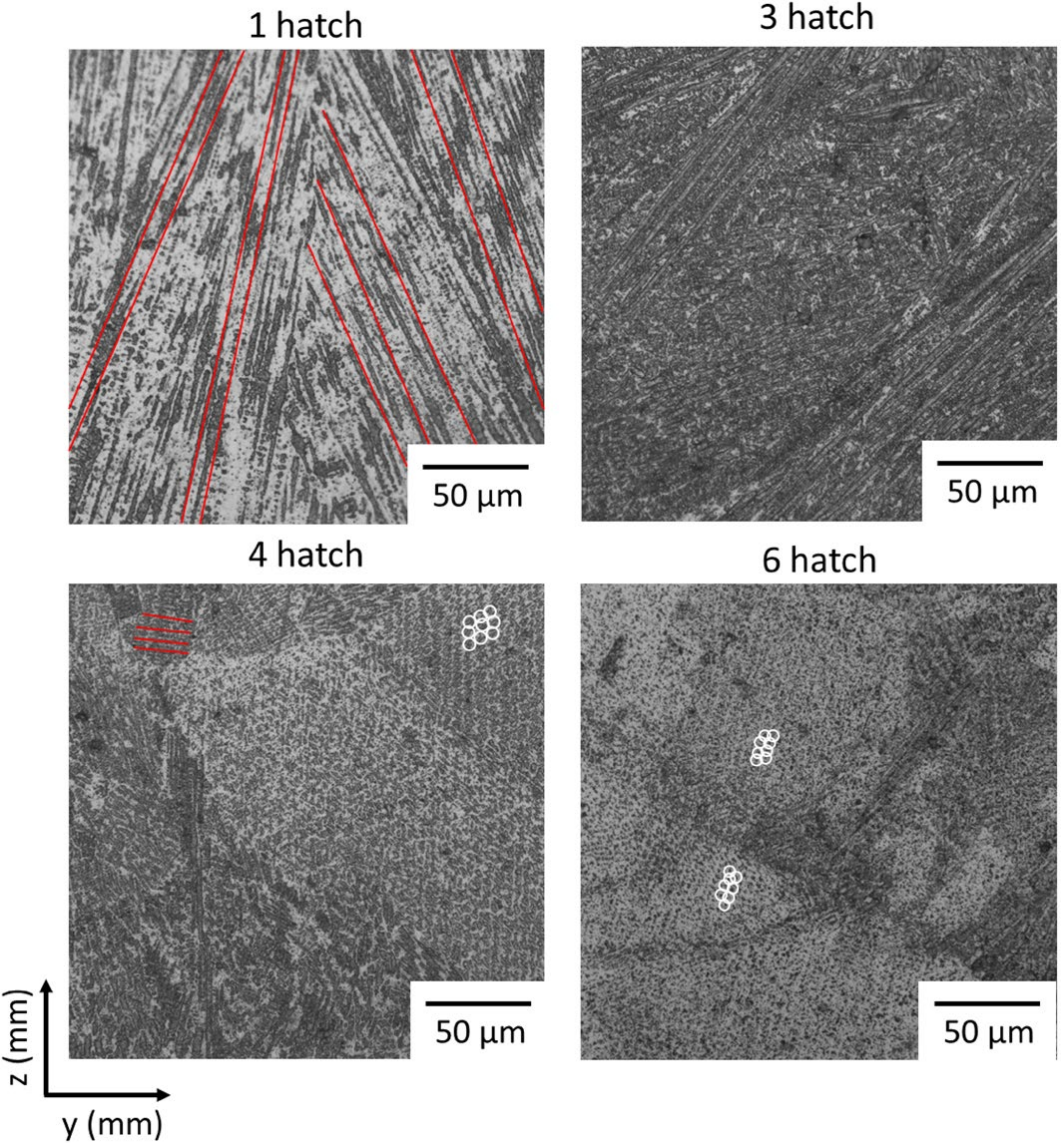
High resolution optical micrographs of 4 walls of varying thickness. Some examples of rod-like sections and end-on sections highlighted (red and white, respectively).

Representative optical micrographs of the YZ section of the walls of varying thickness are shown in Fig. 5; the cellular/dendritic nature of the structure can be seen, these are likely cellular dendrites but will be referred to as dendrites for simplicity. If the dendrites grew in the YZ plane, they would appear as long rod-like structures, if they were perpendicular to the plane, then they appear as dots, if they are angled from the plane, then they would appear as short rods. In the 1 hatch wall, long rod-like structures can be seen throughout; in the 3 hatch wall has a roughly 50–50 split of shorter rod-like sections and end-on/short sections. In 4 and 6 hatch walls, most of the structures are seen end on, with some small sections of elongated dendrites (some examples of rod-like and end-on dendrites are highlighted).

Despite the melt pools being coaxially comparable, the melt pool depth varied by a factor of 2.5×. In samples wider than 2 mm, conduction became more significant, moving the solidification direction out of the YZ plane (as seen by the move away from rod-like dendrites). The change in dendrite orientation is measured in the grain orientation, causing the change from <100> orientation to < 112 > orientation (an X component in dendrite growth was introduced, resulting in a grain orientation with an X component). Additionally, the smaller melt pool in these wider walls has an effect of increasing cooling rate, promoting columnar grain growth. These microstructural differences can have a profound influence on the creep life, crack propagation and tensile properties of the final component^33–35^. Since these microstructural changes could cause the mechanical properties to be insufficient for industrial requirements, this change in structure with thickness must either be accounted for in design or rectified on a component level. This could include adapting the geometry to remove thin sections, or to thicken the geometry and require more machining post-process.

Coaxial melt pool analysis can be used for general melt pool monitoring, producing components, whose melt pool morphology (coaxially) was similar. However, the sharp transition in microstructure with width has revealed that a coaxial view might be insufficient, as it cannot determine the melt pool depth. The final microstructure is dependent on the full thermal field and the 3D geometry of the melt pool, which cannot be fully captured using coaxial monitoring; the melt pool depth is rarely measured and its importance overlooked. It was, however, shown that at a thickness of above ~ 2 mm, the thermal conditions stabilise, resulting in a bulk component, as proposed in literature^28,36^. This change in thermal state from thin walled to bulk (as demonstrated using the Peclet number) is not something which can be easily accounted for by changing processing parameters.

The cost of the setup in these experiments was purposefully kept low, to prove that process control can be achieved without great expense. The frame rate of the camera was limited to 75 fps and the control was only applied at 6 Hz as a way of reducing computational load. Despite the limitations of coaxial monitoring and this control system, it was demonstrated that the microstructural variation within components can be significantly reduced.

### Hardness analysis

**Figure 6:**
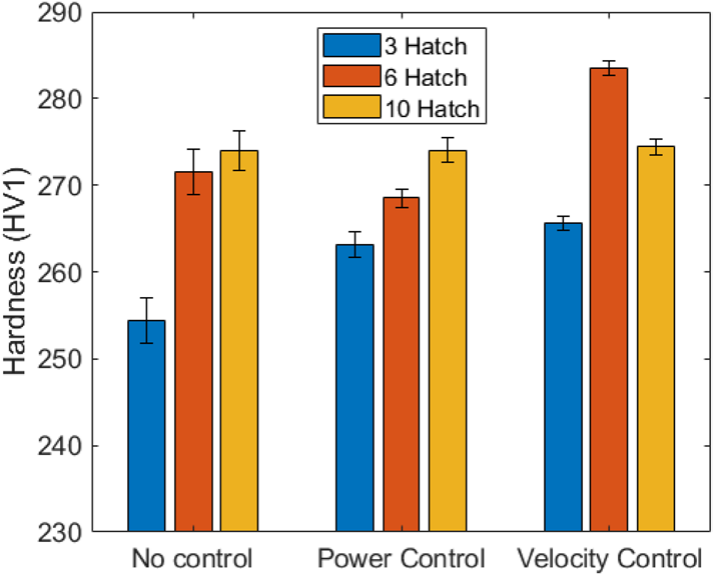
Hardness of walls with no control, with power control and velocity control; standard errors shown.

Average hardness values for the 9 walls are summarised in Table 2 and Fig. 6 along with their associated standard errors; the changes in hardness were not found to correlate with changes in grain structure. For 3 hatch walls, both power and velocity control were found to increase the average hardness, and to slightly decrease the standard error. For the 6 hatch walls, power control slightly decreased the hardness, whilst velocity control slightly increased it; both showed decreased standard error when compared to the no control wall. In the 10 hatch wall, there was minimal change in hardness when power/velocity control were implemented, with standard errors increasing by a small amount.

**Table 2:** Average hardness values for control walls, standard errors included.

Hatches	Average hardness, HV1 (standard error)		
	No control	Power control	Velocity control
3	254.4 (2.7)	263.1 (2.6)	265.5 (2.3)
6	271.5 (1.5)	268.5 (1.1)	283.5 (1.4)
10	274.0 (0.8)	274.0 (0.8)	274.4 (0.9)
Combined	271.3 (0.8)	271.3 (0.7)	276.2 (0.9)

In Fig. 6, it can be seen that the standard errors are highest in the no control (N) samples; however, using a test of two variances (α = 0.05), it was shown that the changes in hardness variability were not statistically significant in the controlled walls. For each control method, the overall average hardness values (combined for the three walls) are also shown and there is no statistically significant change in their variances either. The change in average hardness, however, was statistically significant in three cases, 3P, 3V and 6V (and for the velocity controlled walls as a whole). This suggests that hardness in narrower walls is more sensitive to processing conditions than in wider walls. Since velocity control causes a larger hardness change in the 3 hatch walls (Table 2) and causes a significant hardness change when the hardness’s of the three walls are combined, velocity control is shown to have a greater impact on hardness than power control. Precipitation within the components can be related to the geometry/scan strategy as well as the local reheating^37^, this could explain the remnant hardness variability in the process control components.

## Conclusion

In conclusion, it has been shown that a versatile low cost process control system can be retro-fitted onto an off-the-shelf L-DED machine. Both power and velocity control were shown to control the melt pool morphology, with power control bringing the thermal intensity closer to the target value that velocity control. Variability in both grain area and texture (maximum mud) was reduced using process control by up to 94% for power control (75% in the case of velocity control).

These effects were more significant in narrower components (< 2 mm wide), as narrow components experienced greater variability when built with no control; reduction in the microstructure variability (the combination of grain area and texture is taken to be representative of the overall microstructure) was, however, still present in bulk sections (10 hatches). Velocity control affected the component hardness more than power control, with a slight reduction in hardness variability measured, but this effect was not statistically significant. It is interesting to note that despite power control resulting in the more consistent melt pool morphology, velocity control resulted in the more consistent microstructure.

The stark difference in grain orientation between narrow and wide walls (over 2 mm width) was retained even with process control; this was shown to be due to a change in the heat conduction mechanism and the melt pool depth, which cannot be measured or controlled using coaxial monitoring. This change in grain orientation is expected to have a profound effect on the mechanical properties of the component, especially creep and fatigue properties, which are not captured using hardness measurements.

Despite these limitations, a reduction in grain structure variability was observed due to process control, especially in narrow components; the cost of this solution was a fraction of the cost of many thermal cameras (the cost of the camera used in this work is ~ $400, at least an order of magnitude less than commonly used thermal cameras). An increase in microstructural homogeneity was demonstrated for simple components, with the greatest effect in narrow components. The control scripts are build-shape agnostic, these could be implemented on complex geometries, with the greatest microstructural homogenisation expected in the narrowest sections. Feed forward control could be developed by running process control and then using the determined processing parameters for future builds. Since this process control methodology is versatile, other manufacturing systems could be retrofitted with similar process control systems by using the code that is now publically available; it is hoped that this demonstration of process control will encourage others to implement similar systems, improving the consistency of their processes.

## Methods

### Manufacturing and monitoring

Samples were printed using a BeAM Magic 2.0 L-DED machine using Inconel 718 powder (45–100 µm size range, supplied by LPW) on Inconel 718 substrates with a 3.5 mm work offset. This machine is based on a CNC machine, using a Siemens Sinumerik 840D controller, modified to allow for material addition rather than conventional subtractive manufacturing; this foundation is common for L-DED machines. A 2 kW IPG-2000 ytterbium laser system (1070 nm) was used with a total argon flow of 12 l/min flowing through the nozzle.

Coaxial melt pool monitoring was performed using a greyscale Basler acA1440–73gm camera (costing ~ $400), filtered to a 660–1000 nm wavelength range. A Precitec YW52 welding head with a coaxial camera mount was used. Images were recorded at 75 fps using an exposure time of 4000 µs. The resultant 12-bit images (500 × 500 px, where 1 px = 5.4 µm) were analysed in MATLAB R2021b (Mathworks Inc). For each image, all the pixels were summed to give an overall thermal intensity as previously reported^23,38^; this is a sum of pixel digital levels, so is unitless. This was compared to both the maximum intensity in a single image and to the melt pool area, both showing strong correlation below a thermal intensity of 0.2 × 10^7^^37^, below which, the laser power was ramping at the ends of each hatch. As such, for further analysis, any thermal intensity values below 0.2 × 10^7^ were excluded.

A process control system was developed, adjusting the power/velocity of the machine to keep the thermal intensity near the target, an adapted version reported previously^39^. Every 0.15 s, the last 5 thermal intensities above 0.2 × 10^7^ were averaged. This average thermal intensity was compared to the target of 0.9 × 10^7^ and the power/velocity was adjusted accordingly. Since many L-DED systems are based on Sinumerik controllers, the process control code has been made publically available with the methodology further explained, so it can be adapted to the readers’ need^40^.

The control algorithm was purposefully made to be as simple as possible, so a linear adjustment of power/velocity was used, depending on the measured value of the thermal intensity. The ratio (r) of the current thermal intensity to the target is taken (2 = double the intensity, 1 = equal intensity). The following equation was used to calculate the required adjustment: SF = − mr + c, where SF is the scaling factor; a scaling factor of 1.2 would mean that the power/velocity would be increased by 20% from the original value. m was found to be 0.063 and 0.68 (power and velocity control respectively); c was found to be 1.06 and 0.32 (power and velocity control respectively). These were calculated from four test builds (3 hatches wide), the average intensities of which are shown in Table 3. The further from the target the thermal intensity, the larger the processing parameter correction. For power control, the scaling factor is limited between 0.5 and 1.5, for velocity control, it is limited between 0.2 and 2. The value of m is much smaller for power control meaning that a small change in power has a similar effect as a larger change in velocity i.e. the process is more sensitive to power than to velocity.

**Table 3:** Average thermal intensities for provisional builds.

Power (W)	Velocity (mm/min)	Average thermal intensity
250	2000	3.49 × 10^6^
375	2000	2.04 × 10^7^
250	3000	1.65 × 10^6^
375	3000	1.12 × 10^7^

To measure the effect of process control, three different thicknesses of wall (30 mm long, 15 mm tall) were printed; 3, 6 and 10 hatches wide (roughly 1.5 mm, 3 mm and 4.5 mm respectively), with a bidirectional scan strategy. Each of these widths was printed using constant processing parameters (no control, sample names appended with “N”), with the power being controlled in response to thermal intensity (power control, sample names appended with “P”) and with the velocity being controlled in response to the thermal intensity (velocity control, sample names appended with “V”). The initial processing parameters are shown in Table 4.

**Table 4:** Processing parameters used for control walls; initial processing parameters are shown, control alters the power/velocity parameters respectively.

Sample	Width	Control	Sample Width (mm)	Power (W)	Velocity (mm/min)	Hatch Spacing (µm)	Z Step (µm)	Mass flow (g/min)
3N	3 hatches	No control	1.6	300	2000	400	200	6–7
3P	3 hatches	Power control	1.6					
3V	3 hatches	Velocity control	1.4					
6N	6 hatches	No control	2.7					
6P	6 hatches	Power control	2.7					
6V	6 hatches	Velocity control	2.7					
10N	10 hatches	No control	4.3					
10P	10 hatches	Power control	4.3					
10V	10 hatches	Velocity control	4.4					

Additionally, 6 walls of varying thickness (increasing numbers of hatches) were built to explain the microstructural differences experienced between narrow and wide walls. These walls were 20 mm long, 10 mm tall. These were all built with identical, constant parameters as summarised in Table 1. All walls were sectioned at the midpoint in X in the YZ section, where X is the laser scanning direction, Y is the perpendicular direction in the build plane and Z is the build direction. The widths of all walls were measured optically (YZ secion) and the average of 3 measurements is presented in Tables 1 and 4.

### Microstructural analysis

EBSD of the walls was performed using a Jeol 7900F with an Oxford Instruments Symmetry EBSD detector using a 13 mm work offset and a ~ 90 nA probe current. EBSD analysis was performed using MTEX 5.7.0, an open source MATLAB Toolbox^41^. Grains were calculated using a threshold grain boundary misorientation of 10°, with a minimum of 3 pixels per grain. For each sample, both an average grain area and an average maximum mud were calculated.

Both grain area and maximum mud were given as area-weighted averages. The area-weighted average of a property, $\overline{P}$, can be calculated using:

$$\overline{P}_{area - weighted} = \frac{{\mathop \sum \nolimits_{1}^{N} P_{i} A_{i} }}{{\mathop \sum \nolimits_{1}^{N} A_{i} }}$$

where P is the property being averaged, N is the number of grains, A is the grain area and i is the grain index. The logic behind this is that the effect of a large grain on the final properties is larger than that of a small grain. If a sample has 2 grains, one 1 µm^2^, the other 99 µm^2^, then 99% of the area is taken up by the large grain, so their properties will not contribute equally, hence a grain area weighting was chosen. A similar approach was taken by Lehto et al.^42^ but using grain volumes (e.g. assuming spherical grains); in this case, this would introduce an additional source of error as the grains shapes in AM are strongly anisotropic. Because of the directional columnar nature of many larger grains, it was decided to plot grain area rather than grain size (for which an equivalent grain diameter is normally reported). The calculation of grain size assumes a spherical grain shape, so by stating a grain area we are avoiding using this invalid approximation. For each region, the range of grain areas is simply the area of the smallest grain subtracted from the area of the largest grain.

For each of the walls, one half was polished to a 1 µm finish, these were used for hardness indentation. The other half was further polished using 0.25 µm colloidal silica in preparation for EBSD (scanned using a 3.5 µm step size for control walls, and a 3 µm step size for the walls of varying thickness). The control walls were scanned with the area covering 1 mm (Y axis) in the central section of the sample (Y axis) with a height of 12 mm from the baseplate (Z axis). The walls of varying thickness were scanned with the area covering the full thickness (Y axis) with a height of 1.5 mm (Z axis).

For each of the 9 control walls, the EBSD map was split into 3 mm tall quadrants to quantify the microstructural changes with distance from baseplate. For each of these quadrants, the area-weighted average grain area and maximum mud were calculated. Since the aim of control was to improve microstructural homogeneity, a reduction in grain structure variability was hypothesised, where variability was reported as the range in the quadrant averages.

The walls of increasing hatch number were re-polished and etched with Kallings Reagent #2. Low resolution optical micrographs were taken allowing for melt pool dimensions to be measured. Melt pool dimensions were measured from the top row of melt pools, as these were not remelted by subsequent layers. High resolution optical micrographs were taken to allow for measurement of primary dendrite arm spacing (PDAS), measurements were manually taken in a variety of locations in each sample and averaged.

### Dimensionless numbers

Dimensionless numbers can be used to compare processes with varying conditions and see their effect on the overall process. The main dimensionless numbers applicable to AM are^30^:

- **Fourier Number**: the ratio of diffusive heat rate to storage rate, $F = \frac{\alpha t}{{L^{2} }}$
- **Peclet Number**: the ratio of convection to conduction, $Pe = \frac{vL}{\alpha }$

where α is the thermal diffusivity (2.87 × 10^–6^ m^2^/s for Inconel 718^43^); t is a characteristic time; L is a characteristic length and v is a characteristic velocity. In this work, the Peclet number was calculated for the walls of varying thickness. The laser velocity was used as the characteristic velocity; the melt pool depth was used as the characteristic length.

A higher Fourier number shows that heat diffuses away quicker than it can accumulate^44^, this can have advantages e.g. less potential for distortion^31^. Different characteristic parameters have been used in literature, melt pool length was used as the characteristic length for both the Fourier and Peclet numbers by Mukherjee et al.^31^. In this case, characteristic time can be defined as characteristic length/characteristic velocity—resulting in, $F=\frac{\alpha }{vL}$, the reciprocal of the Peclet number. A higher Fourier number is said to be desirable due to minimised distortion, which means that a lower Peclet number is desirable; this equates to saying that a conduction dominant process is desirable^31^.

It is shown that the Peclet number for builds which result in high density conditions is much larger than that for builds which result in low density conditions. Despite similar melt pool shapes (coaxially), melt pool depth can vary significantly; higher energy density lead to more convection and a deeper melt pool^45^.

### Hardness analysis

Hardness was performed using an EMCO-Test Durascan 70 hardness indenter with a 1 kg load, 15 s hold and the indent automatically measured using a 40× optical lens. Control walls were indented with an array of indents in the YZ section, spaced 0.5 mm in the Y axis and 1.0 mm in the Z axis. Hardness values at sample edges can be unrepresentative, so a row of hardness indents was discarded from each sample edge. For each set of hardness’s, tests of two variances were performed to see whether the process control decreased the variance of hardness (α = 0.05); additionally, two sample t-tests were performed to determine whether the hardness values were significantly different (α = 0.05).

## Data Availability

Data supporting this publication can be freely downloaded from https://doi.org/10.5281/zenodo.7303954 under the terms of the Creative Commons Attribution (CC BY) licence.

## Code availability

The code used in this publication can be freely downloaded from: https://doi.org/10.5281/zenodo.7234173 under the terms of the Creative Commons Attribution (CC BY) licence.
